# HK2: a potential regulator of osteoarthritis via glycolytic and non-glycolytic pathways

**DOI:** 10.1186/s12964-022-00943-y

**Published:** 2022-08-30

**Authors:** Chuncha Bao, Siyi Zhu, Kangping Song, Chengqi He

**Affiliations:** 1 Department of Rehabilitation Medicine, West China Hospital, Sichuan University, Chengdu, 610041 Sichuan People’s Republic of China; 2 Sichuan Key Laboratory of Rehabilitation Medicine, West China Hospital, Sichuan University, Chengdu, People’s Republic of China

**Keywords:** Osteoarthritis, Glycolysis, Hexokinase 2, Chondrocyte, Metabolism

## Abstract

Osteoarthritis (OA) is an age-related chronic degenerative joint disease where the main characteristics include progressive degeneration of cartilage, varying degrees of synovitis, and periarticular osteogenesis. However, the underlying factors involved in OA pathogenesis remain elusive which has resulted in poor clinical treatment effect. Recently, glucose metabolism changes provide a new perspective on the pathogenesis of OA. Under the stimulation of external environment, the metabolic pathway of chondrocytes tends to change from oxidative phosphorylation (OXPHOS) to aerobic glycolysis. Previous studies have demonstrated that glycolysis of synovial tissue is increased in OA. The hexokinase (HK) is the first rate limiting enzyme in aerobic glycolysis, participating and catalyzing the main pathway of glucose utilization. An isoform of HKs, HK2 is considered to be a key regulator of glucose metabolism, promotes the transformation of glycolysis from OXPHOS to aerobic glycolysis. Moreover, the expression level of HK2 in OA synovial tissue (FLS) was higher than that in control group, which indicated the potential therapeutic effect of HK2 in OA. However, there is no summary to help us understand the potential therapeutic role of glucose metabolism in OA. Therefore, this review focuses on the properties of HK2 and existing research concerning HK2 and OA. We also highlight the potential role and mechanism of HK2 in OA.

**Video abstract**

**Supplementary Information:**

The online version contains supplementary material available at 10.1186/s12964-022-00943-y.

## Background

As an important part of skeletal muscle diseases, osteoarthritis (OA) has been a great concern. It is a chronic degenerative bone and joint disease, which can involve in multiple joints (e.g., shoulder joint, hip joint, knee joint) [1, 2]. OA is characterized by progressive degeneration of cartilage, varying degrees of synovitis, and periarticular osteogenesis, including osteophyte formation and subchondral osteosclerosis [3], and the mainly clinical manifestations are pain and joint dysfunction [4, 5].There are many risk factors that affect the occurrence and development of OA, the main risk factors were aging [6], obesity [7], genetic susceptibility, metabolic, traumatic, inflammatory. In addition, biomechanical and epigenetic factors were also included [8]. Currently, the global epidemiological survey showed that as an important part of musculoskeletal disorders, the incidence rate of OA has been increasing gradually since 1990 [9, 10], which seriously affected the quality of life for elderly patients. However, the research on OA mainly is focused on mitochondrial dysfunction, oxidative stress and so on. At present, there are few studies on the pathogenesis of OA from the perspective of glucose metabolism.

Metabolic flexibility is significantly impaired in the OA process, including glucose metabolism [11, 12]. Glucose is metabolized to produce ATP, which is the main energy source for many cellular processes [13]. Glucose metabolism includes glycolysis, pentose phosphate pathway (PPP) and tricarboxylic acid (TCA) cycle [13]. Glycolysis is a strictly regulated process, in which a variety of enzymes (e.g., hexokinase, pyruvate kinase) play an important role in this process [14]. Manoj et al. [15] treated primary chondrocytes with IL-1β, and found that the activity and expression of lactate dehydrogenase (LDH) increased significantly. Results from mRNA sequencing showed that IL-1β induction caused a significant increase in genes expression involved in glycolysis such as pyruvate kinase (PKM), lactate dehydrogenase (LDHA) and hexokinase-2 (HK2) [15]. To determine the function of PKM2 on human OA chondrocyte glycolysis, a study reported that PKM2 was increased in OA chondrocytes compared with healthy control [16]. Furthermore, the active LDHA and lactate (LA) level in synovial fluid of TMJOA patients, were significantly higher compared to healthy control [17], suggesting that glycolysis plays an important role in the progression of OA.

The hexokinase (HK) is the first rate limiting enzyme in aerobic glycolysis, which can catalyze the conversion of glucose to glucose-6-phosphate (G-6-P). Subsequently, G-6-P initiated the main pathway of glucose utilization, including glycolysis, pentose phosphate pathway and oxidative phosphorylation pathway (OXPHOS). Therefore, HK is considered to be a key regulator of glucose metabolism [18, 19]. Hexokinase 2 (HK2) is the isoform of HKs, and more effective than others in promoting aerobic glycolysis [20]. Previous studies provided that overexpression of HK2 in various tumor cells can promote the transformation of glycolysis from OXPHOS to aerobic glycolysis or Warburg effect, which leads to increased glucose uptake and production of LA [21–24]. On the contrary, HK2 silencing could inhibit glycolysis and induce oxidative phosphorylation in hepatocellular carcinoma, and synergistically inhibit the growth of mouse tumor cells with sorafenib [25]. Glucose metabolism seems to be significantly increased in patients with arthritis. An important research question that needs to be addressed is how does glucose metabolism occur during OA initiation and progression? Overexpression of HK2 induced an increase of RNA expression levels of the pro-inflammatory cytokine such as IL-6, IL-8 and metalloproteinases (MMP) in OA FLS [26], which indicated the potential therapeutic effect of HK2 in OA. However, there is no summary to help us understand the potential therapeutic role of glucose metabolism in OA, as well as the relationship between HK2 and pathophysiology of OA remains unclear. Therefore, this review focuses on the properties of HK2 and existing research concerning HK2 and OA. We also highlight the potential role and mechanism of HK2 in OA.

## Biological characteristics of HK2

**Fig. 1:**
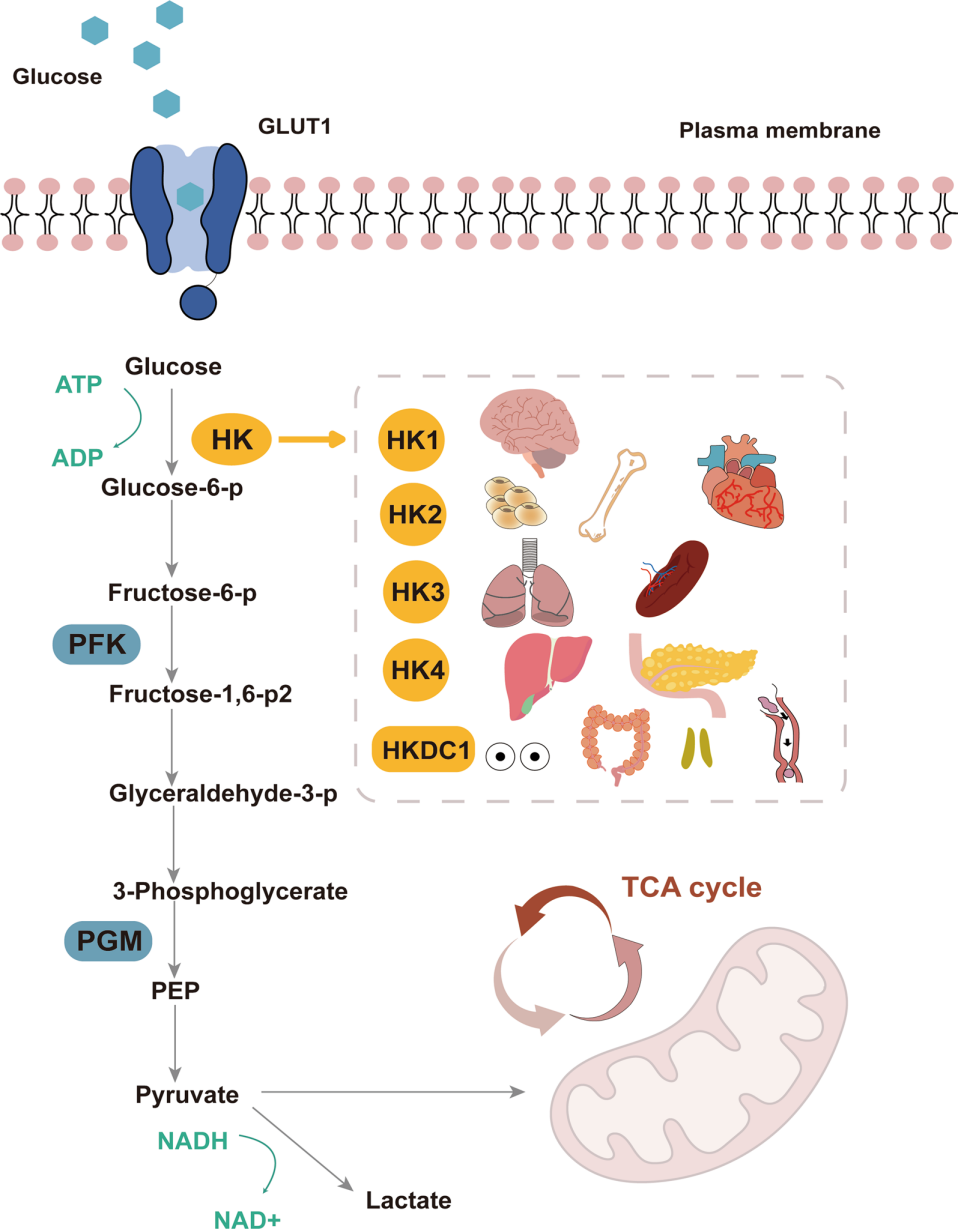
The pivotal role of HK2 in the glycolytic pathway. HK2 can catalyze the conversion of glucose to glucose-6-phosphate (G-6-P). Subsequently, G-6-P initiated the main pathway of glucose utilization, including glycolysis. There are five isotypes of HK family are founded in mammals: HK1, HK2, HK3, HK4 and HKDC1, the expression levels in various tissues and cells are different. HK1 is ubiquitous in mammalian tissues and has a high content in the brain. HK2 is a major regulated isoform in various types of tissues cell lines, and mainly found to be expressed in musculoskeletal system and heart cells. HK3 was mainly distributed in bone marrow, lung and spleen. HK4 regulates insulin secretion, glucose uptake, glycogen synthesis and decomposition in liver. HKDC1 is widely expressed in the pharynx, thymus, colon, and eyes

Hexokinase (HK) is a tissue-specific isoenzyme, catalyzes the first step of glucose metabolism, which is considered to be a key regulator of glucose metabolism [27, 28]. It's worth noting that the results of whole cancer analysis in multiple databases based on the Cancer Genome Atlas (TCGA) showed that abnormal expression of HK family genes was closely related to anomalous amplification, promoter hypermethylation and transcriptional activation [29]. There are five isotypes of HK family are founded in mammals: HK1, HK2, HK3, GCK (glucokinase, HK4) and HKDC1 (hexokinase domain contains 1), the expression levels in various tissues and cells are different [30, 31]. HK1 is ubiquitous in mammalian tissues and has a high content in the brain, which is called "brain hexokinase". HK1 is composed of N-terminal regulatory and C-terminal catalytic domains [32, 33]. A recent study demonstrated that HK1 is closely related to the activation of inflammation in nervous system diseases such as Alzheimer's disease (AD) [33]. HK2 is a major regulated isoform in various types of tissues cell lines, and mainly found to be expressed in musculoskeletal system and heart cells [34, 35] (Fig. 1). HK3 was mainly distributed in bone marrow, lung and spleen [35]. HK4 regulates insulin secretion, glucose uptake, glycogen synthesis and decomposition in liver, called “glucokinase” [27]. Interestingly, a novel HK-like gene called hexokinase domain containing protein-1 (HKDC1) was recently uncovered, it is the same as the other four HKs, the only difference is the last eight amino acids of C-terminal [29]. HK4 has only one kinase structural active site domain, differently, there are two kinase structural active site domains in HK1 and HK3. HK1 and HK3 have two kinase structural active site domain (N-terminal structural site domain without enzymatic activity and a C-terminal active site domain with enzymatic activity), but unlike HK1,HK3 and HK4, HK2 has high affinity for glucose, both N-terminal and C-terminal of which have catalytic activity [27, 36]. The molecular weight of HK2 protein is about 100 kDa, which is produced by replication and tandem ligation of precursor genes similar to GCK. In addition, the N-terminal and C-terminal of HK2 are sensitive to the inhibition of G-6-P, but the C-terminal of HK2 is significantly inhibited when the concentration of G-6-P is much higher than that of N-terminal [36]. These special kinetic characteristics may make HK2 play a unique role in glucose metabolism under various physiological conditions.

## Distinctive glycolytic function of HK2

Cells under dynamic conditions (e.g., hypoxia, oxidative stress, pathological changes) need to undergo metabolic changes in order to maintain their growth, such as glucose metabolism [37, 38]. Previous studies have confirmed that HK2 plays the pivotal role in metabolic recombination [39], which is induced by carcinogens or hypoxia [39–41]. HK2 has an N-terminal active site domain (A conservative short hydrophobicity α Helical domain), which could bind to mitochondrial outer membrane voltage-dependent anion channel 1 (VDAC1) protein [42]. The binding of HK2 and VDAC1 provides a variety of "kinetic advantages" to promote hexokinase reaction, reducing the sensitivity of HK2 to the product of G-6-P, and thus increasing its affinity for ATP [43–45].

Moreover, the HK2 combined with mitochondrial VDAC can take advantage of ATP produced by mitochondria to promote glucose into cells and promote glycolysis [46]. HK1 and HK2 have two equivalent domains of glucokinase, but distinct from HK1, HK2 has enzyme catalytic activity [47], potentially a promising target to positively regulate metabolism in cells, especially musculoskeletal cells under metabolic changes due to physiological or pathological conditions (Fig. 1). Furthermore, the analysis of HK2 binding to mitochondria demonstrated that HK2 located at mitochondria-endoplasmic reticulum (ER) contact sites, which called MAMs (mitochondria-associated membranes), was found to be involved in several metabolic pathway in different types of cells [37, 48].

## Non glycolytic function of HK2

HK2 not only has an enzymatic function that phosphorylating of glucose to G-6-P, but also has non-glycolytic function. Studies have shown that one of the non-enzymatic functions of HK2 is directly regulate cell apoptosis, and it is considered as a potential target to prevent organelle damage and maintain cell viability after apoptosis. Specifically, HK2 seems to interfere with BCL2 family members that mediate mitochondrial membrane damage [49, 50]. The research showed that recombinant Akt phosphorylated HK2 and inhibited the release of cytochrome C in mitochondria of adult mouse heart induced by Ca2^+^. Akt can increase the activity of mitochondrial HK2 and cause changes in the expression of apoptotic proteins, such as Bax and Bak [51]. However, the molecular mechanism of HK2 against apoptosis is still unclear. Besides, HK2 plays an important role in transcriptional regulation by binding to transcriptional regulated metabolic enzymes [52]. Therefore, HK2 may be directly or indirectly involved in the transcriptional activation, signal transduction or phosphorylation of some signaling molecules. The specific mechanism needs to be further explored, especially in musculoskeletal tissues.

## Existing evidence concerning HK2 and OA

Aerobic glycolysis refers to certain cells, including many rapidly proliferating cells, which show high fermentation rate even in the case of sufficient oxygen. This is a metabolic phenotype, which is a hot spot in the field of cancer research, but this phenotype is not unique to cancer [53, 54]. HK2 was confirmed to be expressed in RA and OA-FLS (fibroblast synovial -like cell lines), TNF and hypoxia could increase the HK2 protein level in OA FLS, meanwhile, overexpression of HK2 increases the RNA levels of pro-inflammatory cytokines such as IL-6, IL-8 and MMP in OA FLS cell lines [26]. In addition, TGF-β1, an important regulator of cartilage homeostasis, induces the increase of aerobic glycolysis flux and the decrease of oxidative phosphorylation in human articular chondrocytes (HACs) in OA. After 48–72 h treatment with TGF-β1, the expression of GLUT1 and HK2 two times higher than that in control. Interestingly, another hexokinase isoform HKI was also increased in the treated cells [55]. A present study with a focus on HK2 validated that the expression of HK2 was increased in peripheral blood mononuclear cells (PBMCs) compared with that in HCs examined by real-time PCR in an RA and OA cohort [56]. These findings highlight that HK2 may be involved in the pathogenesis of OA. Further exploration on the exact role and mechanism of HK2 in the pathogenesis of OA is of great significance to find metabolic targets in treatment development for OA.

## Potential role and mechanism of HK2 in OA

HK2 is an important kinase in glycolysis, that prime glucose, whose functions in autophagy regulation, cell death and other physiological and pathological state. However, the function and mechanism of HK2 in OA development are still unclear. Chondrocytes have unique characteristics of metabolism and synthesis especially under hypoxia condition. Therefore, researches on the role of HK2 in glycolysis and the potential underlying mechanisms would help provide helpful insights into the understanding of the potential link between HK2 and OA metabolism.

### Glycolysis function in OA

In the glycolysis reaction, there are several important enzymes and processes that affect the glycolytic flux, including HKs, PFKs and LA output. These key enzymes and processes play an important role in the development of chronic degenerative diseases such as OA [16, 57]. The metabolic shift in chondrocytes is critical in the development of OA, in which changes from a state of regulatory rest to an active state of high metabolism [58].

The expression of glycogen protein 1-(GYG1)-asparagine, which is an enzyme involved in the biosynthesis of glycogen, was significantly down-regulated in the metabolomics transcriptome integration analysis of RA and OA, indicating that glucose homeostasis might be implicated in the pathogenesis of OA [59]. Meanwhile, it was also found in several vivo studies that the contents of metabolites related to glycolysis (e.g., alanine, serine and LA) increased significantly in OA [60, 61]. PKM2 is a pivotal regulator in the pathogenesis of OA. A recent study showed that the expression of PKM2 was up-regulated in OA chondrocytes compared with healthy control chondrocytes, PKM2 knockdown can inhibit the proliferation and promote apoptosis of OA chondrocytes, and down regulate the expression levels of COL2A1 and SOX-9 [57]. LDHA has the function of promoting ROS formation in chondrocytes during the inflammatory state, while inhibiting the activity of LDHA was an effective therapeutic target for OA. Manoj et.al found a significant increase in expression of genes involved in glycolysis and fermentation such as HK2 and LDHA, and the level of LA in cell culture supernatant was also increased [15].

Impaired glucose uptake would compromise cell function and potentially result in osteoarthritis [62]. Racid et al. confirmed that both OA FLS and RA FLS showed lower respiratory rate and increased glycolysis activity [63]. On the contrary, using a glycolysis inhibitor 2-deoxy-d-glucose (2-DG), the levels of inflammatory markers were significantly decreased, indicating that glucose metabolism plays an important role in FLS metabolism and is crucial to the pathogenic function of FLS under pro-inflammatory stimulation [63]. Meanwhile, the GLUT1 expression and the level of LA in FLS of OA patients were increased, and glycolysis block inhibited the migration ability of these cells [63].

These evidences above indicate that glucose metabolism plays an important role in the development of OA. HK2 may be a potential modulator of OA metabolism, which still needs to be further clarified.

### Potential protein kinase targets of HK2 in OA

**Fig. 2:**
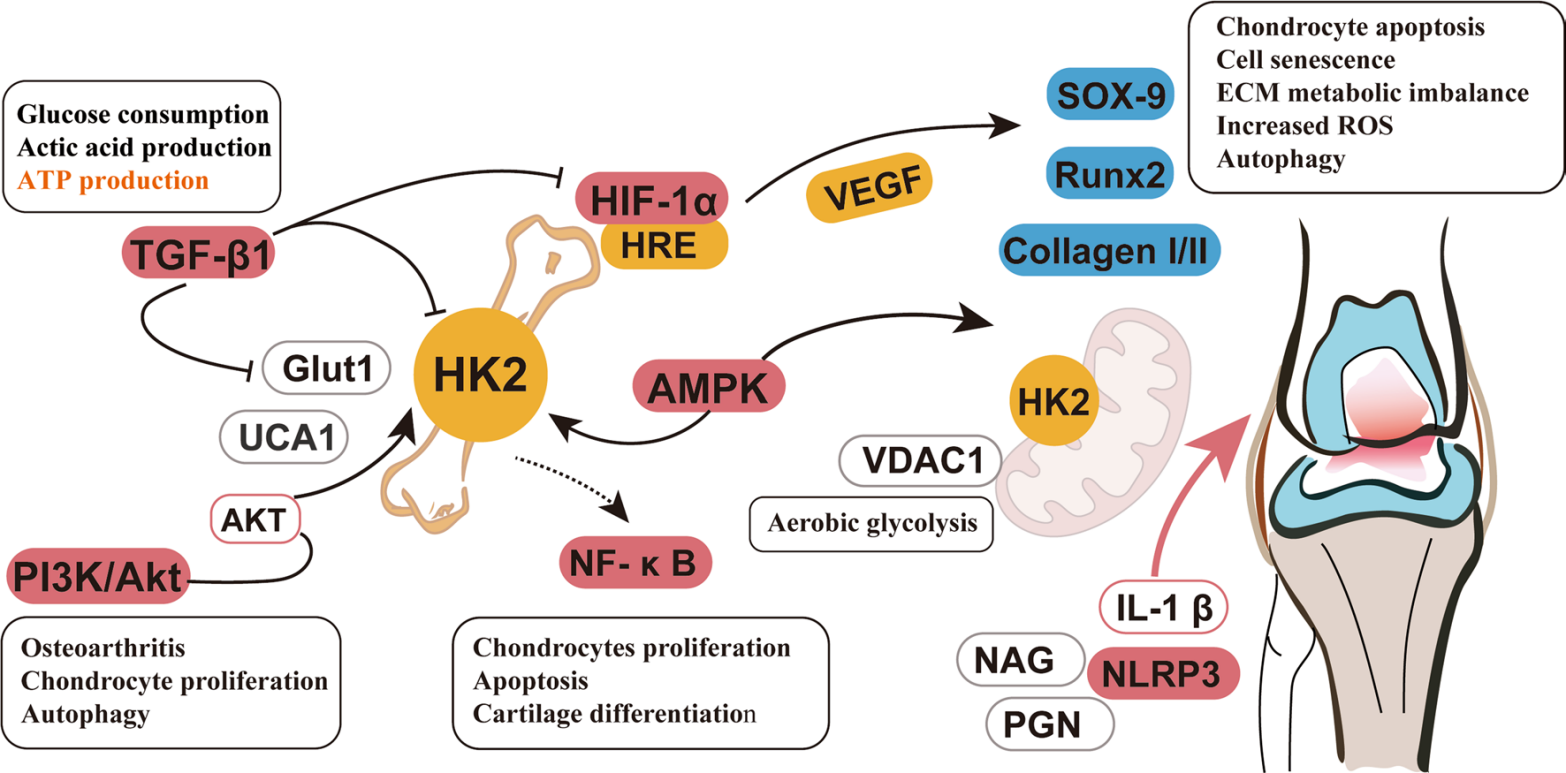
The potential role and mechanism of HK2 in OA. HK2 is an important kinase in glycolysis, where it functions in autophagy regulation, cell death and other physiological and pathological state. The exact role of HK2 and aerobic glycolysis in the pathogenesis of OA also related to the certain pathway, such as HIF-1α, AMPK, TGF-β1, PI3K/Akt, NF-κB, NLRP3 inflammasome

It has been clarified that the HK2 could interact with many protein molecules, but HK2 as a protein kinase in OA progress is barely elaborated. Therefore, we summarize several potential targets of HK2 in OA (Fig. 2).

#### Hypoxia inducible factor-1 (HIF-1)

Hypoxia is the allosteric regulation of glycolysis, and under conditions of hypoxia, most eukaryotic cells can change their main metabolic strategies from mitochondrial respiration towards increasing glycolysis to maintain ATP level [64]. Hypoxia inducible factor-1 (HIF-1) is an important transcription factor and a major regulator of glycolysis, which can up-regulate the transcription and expression of glucose transporters and glycolytic enzymes [64–67]. Furthermore, Steve et al. [68] clarified that HIF-1α signaling in chondrocytes regulates the synthesis and modification of collagen in chondrocytes by inducing metabolic changes. Similarly, increased expression of HIF-1α was detected in human and mouse OA chondrocytes, accompanied by chondrocyte apoptosis, cell senescence, extracellular matrix (ECM) metabolic imbalance, and increased ROS and autophagy [69]. Subsequent experiments showed that knockdown of HIF-1α partially eliminated hypoxia-induced cell damage and played an important role in ROS production [69]. The expression of HIF-1α and VEGF is also elevated in OA cartilage and IL-1β-induced chondrocytes. Conversely, the suppression of HIF-1α could reduce VEGF expression levels, which contributes to the production of collagen II, aggrecan and SOX9, further inhibit the expression of collagen I and RUNX2 [70]. One study reported that the HIF-1α and Runx2 were increased in the chondrocytes from both OA and IL-1β conditions. When HIF-1α was silenced, the glycolytic metabolism of chondrocytes was also suppressed, suggesting that HIF-1α plays a role in the self-repair of the glycolytic metabolism of OA [71]. Therefore, from the evidence above, it is worth noting that the HIF-1α may become a potential target of HK2 in chondrocyte metabolism, and further studies on that should be conducted to confirm the critical role.

#### AMPK

Cells need to constantly adjust their metabolic pathways to meet their energy requirements and respond to nutrient utilization. AMP-activated protein kinase (AMPK) has attracted widespread attention as a potential target for the treatment of diseases related to metabolic disorders [72, 73]. The decrease in AMPK activity was observed in human and mouse OA cartilage tissues, which was achieved by catalyzing the phosphorylation of specific threonine in AMPK (AMPKα1), suggesting that AMPK activity in chondrocytes is the key to maintaining stable state of joint function and development of OA [74, 75]. Besides, attempts to reverse AMPK dysfunction can reduce inflammation and prevent the progression of OA. Yun et al. found that AMPK activator (A-769662) significantly promoted the expression of PGC1 α in OA chondrocytes through SIRT1 signaling and rescued mitochondrial defect [76]. Another study also showed that the AMPK can limit oxidative stress and improve the mtDNA integrity and function of OA chondrocytes via activating SIRT3 [77]. The activation of AMPK is achieved in part by maintaining glycolysis. A recent study reported that blocking the level of IL1β-enhanced MMP13 by using galactose-replacement in osteoarthritic chondrocyte inversely increased bio-markers associated with p-AMPK, further confirmed AMPK is a downstream regulatory molecule in the process of glycolysis in OA [78]. The above studies provide clues to the important role of the correlation between AMPK and aerobic glycolysis in the pathogenesis of OA.

#### Transforming growth factor (TGF-β1)

Transforming growth factor beta1 (TGF-β1) is a multifunctional cytokine, which can regulate cell cycle, growth, development, proliferation and differentiation, ECM synthesis and immune response [79, 80]. Cell metabolism is affected by the micro-environment around, and studies have demonstrated that TGF-β1 inhibits the glycolysis process by inducing natural regulatory T cells, and also significantly down-regulates the expression of key enzymes in the glycolysis pathway (e.g., Glut1, Glucose transporter, HK2 and HIF-1α) [81]. In addition, TGF-β1 stimulation can up-regulate glycolysis in dermal fibroblasts, while inhibition of glycolysis can reduce the pro-fibrosis induced by TGF-β1 [82]. Therefore, TGF-β1 plays a dominant role in metabolic reprogramming (inducing the conversion of oxidative phosphorylation to aerobic glycolysis). It is known from the cellular and molecular level that OA is characterized by a transition from a healthy steady state to a catabolic state, and TGF- β1 signaling plays an important regulative effect in maintaining the morphology of articular cartilage and the internal environment of cartilage cells [55, 58, 83]. Wang et al. showed that in human articular chondrocytes (hAC) with OA, TGF-β1 stimulation increased glucose consumption and LA production, meanwhile reduced ATP production [55]. Under TGF-β1 treatment, Glut1 and HKII significantly increased by over than two times. Interestingly, the expression of another hexokinase isoform, HKI, also increased in cells treated with TGF-β1 [55]. Ni et al. found that the expression of HK1 and HK2 was detected in c-Kit lineage cells, and TGF-β1 significantly increased the expression of HK1 and HK2 in a time-dependent manner, and the activity of HK was also up-regulated, showing that TGF -β1 and HK may have a direct regulatory effect [84]. Findings above provide very important clues to the interaction between TGF-β1 and HK2 in the process of metabolic reprogramming, especially how to promote the pathogenesis of OA.

#### PI3K/Akt pathway

The phosphoinositide 3-kinase (PI3K) signaling pathway plays a key role in cell growth control and glucose metabolism. Akt (also known as protein kinase B) is a serine/threonine protein kinase directly activated by PI3K [85]. The PI3K/Akt pathway can be activated in response to insulin, growth factors and cytokines, thereby regulating a wide range of processes such as glucose metabolism, biosynthesis, and redox balance [86, 87]. Multiple control points (HK2, PFK1 and PFK2) in the glycolysis process are regulated by the PI3K/Akt pathway. Furthermore, AKT also controls the key steps of glycolysis by phosphorylation of specific glycolytic enzymes [85]. AKT activation has been found to promote HK2 activity by increasing the association with the VDAC of the outer mitochondrial membrane [36]. The intracellular localization and kinetic characteristics of HK2 is conducive to the transport and utilization of glucose during glycolysis [88]. In addition, blocking PI3K/Akt pathway inhibits the activity of key enzyme HK2 in aerobic glycolysis and cell proliferation [89]. Similarly, accumulating data have proved that PI3K/Akt can directly regulate the activity of HK2 in cells [90, 91]. The previous analysis showed that compared with healthy cartilage, the expression of phosphorylated PI3K/Akt increased in OA cartilage [92], while the inhibition of PI3K/Akt/mTOR signaling alleviated OA induced joint injury by restoring cartilage homeostasis, enhancing autophagy and inhibiting inflammation [93]. The association of PI3K/Akt and HK2 is potentially critical in the pathogenesis of OA.

#### NF-κB

Nuclear factor kappa B (NF-κB) is a transcription factor, which is ubiquitous in various types of cell lines, and plays a central role in the development of osteoarthritis [94]. One study found that the transient activation of NF-κB in ATDC5 chondrocytes was involved in the regulation of chondrogenic differentiation [95]. As an upstream regulator of the classic NF-κB pathway activation mechanism, IKKs are closely related to the catabolism of chondrocytes. The intra-articular administration of (BMS-345541) IKKs inhibitors significantly suppressed the expression of MMP13 and ADAMTS5, as well as prevented cartilage damage at 8 weeks [96]. Furthermore, another study also suggested that NF-κB signal transduction plays a critical role in the regulation of glycolytic activity, and activates Ca2^+^/NF-κB axis to promote glycolysis and microenvironment remodeling by increasing the production of downstream cytokines [97]. Wang et al. used glycolysis inhibitor 2-deoxyglucose (2-DG) to treat adjuvant arthritis (AA) rats, and found that HK2 expression was positively correlated with synovial hyperplasia, inflammatory cell infiltration and cartilage destruction, which further confirmed that the effect of HK2 glycolysis inhibitor is closely related to the activation and inhibition of NF-κB signaling [98]. The above research indicated that the role of links between HK2 and NF-κB signal transduction pathway in the process of cell metabolism for OA development although critically associated but still remains implicit, further research are required to reveal the accurate association between HK2 and NF-κB signal in OA.

#### NLRP3 inflammasome

Inflammation has long been identified as the driving factor of many chronic diseases and autoimmune disease, including Alzheimer's disease [99], atherosclerosis [100] and OA [101]. The NLRP3 (NLR family, containing three pyridine domains) inflammasome is a multi-component assembly of adaptor and effector proteins highly expressed in myeloid cells, consisting of (NLRP3) NOD-like receptor protein 3, adaptor protein, apoptosis associated speck- like protein (ASC) and caspase-16 [102]. Studies have demonstrated that the N-acetylglucosamine (NAG) is an activator of NLRP3 inflammasome [103]. PGN and NAG can inhibit hexokinase and induce its dissociation from mitochondria outer membranes, thus affecting the metabolism of hexokinase activity conditions to trigger the activation of inflammasomes, which indicates that the breakdown of the specific metabolism of hexokinase function also induce the activation of inflammasomes [102, 104]. There is an important connection between glucose metabolism and the activation of NLRP3 inflammasomes [103]. Ahmad et al. found that ATRA can enhance the expression of HK2 and shift the metabolism from LPS activated by macrophages to glycolysis, leading to the activation of NLRP3 inflammasome [105]. Cartilage destruction and subchondral osteosclerosis were found in OA patients and OA model rats, and the expression level of NLRP3 was significantly increased in synovial tissue [106], which indicates that NLRP3 inflammasome was involved in the pathogenesis of OA [107]. Future studies will further determine the interaction between HK2 and NLRP3 and reveal whether it plays an important role in OA.

## Conclusions

Metabolism is central to maintain the function of cartilage and synovial joint. Under adverse microenvironmental conditions, energy shift to glycolysis plays pivotal roles in the progression of OA. HK2 has been proven to play multiple roles of metabolic enzymes during glycolysis in musculoskeletal tissues including bone and cartilage through the regulation of cell metabolism and many other important cellular activities, such as cell growth, proliferation, survival, autophagy, and apoptosis. Although the understanding of the role of glucose and energy metabolism in OA is incomplete, studies on HK2 have greatly expanded our understanding of the glucometabolic interaction network in the pathogenesis of OA, in which HK2 may act as regulators of transcription factors growth factors, inflammatory factors and autophagy related molecules, which potentially affect ROS level, mitochondria function, extracellular matrix remodeling, and the proliferation and differentiation of chondrocytes and synovial cells. Through investigating key factors HK2 included in aerobic glycolysis, and the regulatory pathways, such as TGF-β1, AMPK, PI3K/Akt, NF-κB, NLRP3, potential mechanisms underlying relationship between the regulation of HK2 and energy metabolism in OA will be further fueled by further investigations (Fig. 2). Potentially, new therapeutic approaches for the treatment of OA or related surrogate outcome will then be developed, especially biomarkers in energy metabolism and small molecular drugs targeting downstream protein or kinase of HK2.

## Abbreviations

OA: Osteoarthritis
OXPHOS: Oxidative phosphorylation
HK2: Hexokinase 2
G-6-P: Glucose-6-phosphate
LA: Lactate
HKDC1: Hexokinase domain contains 1
VDAC1: Voltage-dependent anion channel 1
HIF-1: Hypoxia inducible factor-1
AMPK: AMP-activated protein kinase
TGF-β1: Transforming growth factor
PI3K: Phosphoinositide 3-kinase
NF-κB: Nuclear factor kappa B
NLRP3: NOD-like receptor protein 3
RUNX2: Runt-related transcription factor 2
ECM: Extracellular matrix
